# Chestnut shell aqueous extract as a green efficient corrosion inhibitor for acid-cleaning carbon steel

**DOI:** 10.1371/journal.pone.0340769

**Published:** 2026-02-13

**Authors:** Ceshen Zhou, Taoyu Zhou

**Affiliations:** 1 College of Animal Science and Food Engineering, Jinling Institute of Technology, Nanjing, P.R.China; 2 School of Environmental Science and Engineering, Nanjing Tech University, Nanjing, P.R.China; National Chung Cheng University, Taiwan & Australian Center for Sustainable Development Research and Innovation (ACSDRI), AUSTRALIA

## Abstract

As a new candidate for green corrosion inhibitor as well as a new application of a common food residues for waste resource recycling, an aqueous *extract of chestnut shells (ECS),* from a discarded item *CS* (*chestnut shells*) of a daily food chestnut, is investigated and evaluated using multi-means. For the ECS sample itself, UV-visible absorption result reveals its *π*→π* transition of electrons similar to isoflavonoid; an orthogonal array with four factors is used to optimize the optimal combination of CS dosage, alcohol dosage, temperature, and pH for its preparation, providing the result of “CS 20g/L, ethanol 20% (v/v), 50^o^C, and pH 9”. For the ECS sample used as corrosion inhibitor for carbon steel in 1mol/L HCl solution, the Tafel curve of electrochemical polarization demonstrates its mixed-type corrosion inhibition performance dominated by anodic control. *Electrochemical impedance spectroscopy* (*EIS*) data indicates that a high resistance adsorption barrier film formed by ECS dominates a combined contribution of the film resistance and charge transfer resistance to the corrosion inhibition of carbon steel. Both the Tafel and EIS data achieve an acceptable inhibition efficiency of around 80% for ECS alone at a concentration of 56.6mL/L. Specially important for practical applications, the ECS-induced weight-loss gives an actual-simulated corrosion rate inhibited to around 1.21mm/y, which well meets the 2.23mm/y as limit requirement of the national standard. Furthermore, the ECS-adsorbed process is discussed through adsorption isotherms that follows Temkin model and the model fitting result estimates a change of −27.13kJ/mol in the standard free energy of adsorption, indicating the existence of a mixed process of physi- and chemi-sorption of the ECS on the steel surface. This work highlights the ECS considerable potential for title application namely acid-corrosion inhibition of carbon steel equipment.

## Introduction

Chestnuts, often referred to as “the king of dried fruits”, are a widely consumed natural food. China alone produces an impressive 1.56 million tons of chestnuts annually, accounting for approximately 80% [1] of the global output, with nearly 40-thousand tons exported each year [2]. However, it is regrettable that *chestnut shells* (*CS*), which account for approximately 10% of the total weight of chestnuts, are often discarded as waste. The potential for utilizing CS as a valuable resource remains largely unexplored and warrants further investigation.

In recent years, researchers at home and abroad have explored the physical and biological properties of CS, including its ion adsorption capabilities [3,4], antioxidant, antibacterial and anti-obesity [5,6]. Previous studies have also investigated some applications of CS in the textile dyeing and printing, and the food processing industries [7]. Despite these advancements, there have been no reported studies on CS for use in industrial water treatment, materials protection, or corrosion inhibition.

In daily industrial operations and scheduled maintenance, acidic chemical cleaning of metal equipment is often necessary, and the use of corrosion inhibitor is essential to mitigate the corrosive effects of acidic media on the surface of various metal materials such as steel, copper, zinc, aluminum, etc. Due to its favourable mechanical properties and affordable price, carbon steel is widely present in various industrial equipment and its corrosion inhibition research in hydrochloric acid or sulfuric acid environments is highly valued. It is widely known that applying corrosion inhibitors is still thought to be one of the most popular techniques used to improve steel shielding and corrosion resistance [8]. In the past few years, Jafari *et al* studied the inhibition ability of the aniline violet and benzoyl cyclohexanediamine on carbon steel in 1M HCl solution [9,10]. Kamel *et al* reported numerous studies on corrosion inhibitors for carbon steel in hydrochloric acid environments, e.g., paprika extract, resorcinol derivative, aminotriazole thiol etc [11–15]. Abdallah *et al* confirmed the efficacy of natural mentha oil as a safe and environmentally acceptable anti-corrosion agent for carbon steel in 1M H_2_SO_4_ solutions [16,17]. Khormali *et al* evaluated the efficacy and synergistic effect of oleic imidazoline and binary benzimidazole derivatives as corrosion inhibitors for carbon steel in 1M HCl [18,19]. Wanees *et al* investigated benzylidene compound and modified chitosan as an effective inhibitor against the corrosion of C-steel in 1M HCl solutions [8,20,21]. These published achievements involve the synthesized/derivatized/modified/screened organic compounds, natural products, and some plant extracts as corrosion inhibitors for carbon steel in corrosive hydrochloric acid media. Taken together, their satisfactory results confirmed by experiments, for example in 1M HCl solutions at room temperature, provide general information on most effective concentration of 130 ~ 1980mg/L, maximum inhibition efficiency of 61.4 ~ 98.3% approximately, minimum corrosion rate of 0.17 ~ 2.01mm/y, type of corrosion control, and the surface adsorption corrosion inhibition mechanism of a certain isothermal-fitted model. Among them, the effective concentration of modified chitosan as low as 5mg/L [21] and the inhibitory efficiency of mentha oil as high as 98.3% [16] are impressive.

Of the various corrosion inhibitors, those derived/extracted from natural plants are green, cost-effective, and environmentally friendly. Interestingly, the first patent for a corrosion inhibitor was based on a plant extract [22]. To date, several natural corrosion inhibitors have been studied, derived from the leaves, roots, stems, and residues of various plants [23,24]. Eddy specially reviewed plant wastes as alternative sources of sustainable and green corrosion inhibitors, and further pointed out that plants extracts are good corrosion inhibitors because not only they contain phytochemicals which have atoms with potentials adsorption tendency such as P, O, N, S atoms but also they possess conjugated, aromatic and *π*-electron systems [25].

Inspired by these published interesting literatures, we conducted a preliminary study on an aqueous *extract of chestnut shells (ECS).* This manuscript mainly aims to prepare ECS samples and evaluate their corrosion inhibition performance on carbon steel in a 1mol/L hydrochloric acid solutions, in order to provide potential applications for the title objective. This study employed multifaceted approaches including orthogonal array, UV-vis absorption, Tafel curve, AC impedance spectroscopy, weight loss, as well as adsorption isotherm model for the preparation, analysis, evaluation, and mechanism speculation of the obtained samples.

The application of ECS samples as corrosion inhibitors, particularly in acidic environments, represents a novel investigation. Results demonstrate that ECS alone at apparent concentrations of a few to tens of mL/L, exhibits fairly anti-corrosive effect with acceptable inhibition efficiency as well as low corrosion rate of special significance in practice. Additionally, both the ECS itself and its preparation are environmentally friendly due to being derived from the water-alcohol soluble components of a common food residues and being free of toxic or harmful chemical reagents respectively. As a discarded item from a daily food chestnut, CS used as primary raw material for ECS preparation reduces the demand for plants/foods themselves and especially expensive chemicals, making its production both economically feasible and sustainable in line with the principle of waste resource re-utilization.

## 1 Experimental section

### 1.1 Instruments and reagents

UV-visible Spectrophotometer (M/L6S, INESA anal. instr. Co. Ltd, P.R.China) with 1cm quartz colorimetric cells. Electrochemical instruments (M/CHI660E, CH Instruments Ins., USA; M/PARSTAT 4000, AMETEK Princeton Applied Research, USA). Carbon steel (ϕ5.0mm) as *working electrode* (WE), A3 grade formerly, almost equivalent to the current Q235A grade in P.R.China [26]. SCE (saturated calomel electrode) as *reference electrode* (RE). Platinum wire as *auxiliary electrode* (AE).

Carbon steel hanging sheet (Steel serial A3, *ibid*; Size type II, 72.4 × 11.5 × 2mm^3^; Total surface area of 20.00cm^2^). Rust removal solution for the hanged-up metal (hexamethylenetetramine 8g dissolved in hydrochloric acid solution (1 + 4) 1L). Several chestnut shells (Southern Anhui province, P.R.China). Ethanol anhydrous. NaOH (A.R.). HCl aqueous solution (1mol/L). Pure water (R.O. grade).

### 1.2 Preparation of ECS samples

Accurately weigh a certain amount of CS and mechanically crush them into proper size. The crushed CSs are then immersed in an aqueous solution with some ethanol dosed and pH adjusted by acid/alkali at a controlled temperature. Filtration to obtain a filtrate gives the ECS sample as an aqueous extract. An *L*_9_(3)^4^ orthogonal array is employed to optimize the extraction process including four factors: mass-volume “concentration” (g/L) of CS dispersed in water, ethanol dosage (v%), pH value, and immersing temperature. ECS samples’ absorbance serves as the response variable. In all of subsequent measurements, the apparent concentration of the ECS sample used is expressed in mL/L as a ratio of the used solute volume to the used solutions volume. Of some possible conversions of related concentration units the literature is valuable and referenced [5].

### 1.3 Spectrum and absorbance measurements

Measurements of absorption spectrum and UV-vis absorbance of the ECS sample solutions are conducted using a diluted solution of 25 times in pure water, that is to say that the ECS concentration is 40mL/L by volume ratio, at room temperature (12°C) to perform full wavelength scanning absorption spectra in the 190 ~ 900nm range or to measure the absorbance at a certain wavelength. The reference solution used is shown in the figure.

### 1.4 Electrochemical measurements

Electrochemical measurements are carried out using a three-electrode system with tested solutions at room temperature (5°C). The polarization range of electrochemical Tafel curve is *open circuit potential* (*OCP*) plus or minus 250mV, with a potential sweep rate of 10mV/s. *Electrochemical impedance spectroscopy* (*EIS*) uses a sinusoidal AC signal of a 5mV amplitude with an initial potential of the OCP to performs frequency scanning in range of 1MHz to 0.01Hz. The corrosion *inhibition efficiency* (*IE,%*) of ECS samples is calculated by Equation (1) for Tafel polarization or Equation (2), (3) for EIS results:


$$IE=(i_{corr,0}-i_{corr})/i_{corr,0}\times 100\%$$ (1)

$$IE=(R_{t}-R_{t,0})/R_{t}\times 100\%$$ (2)

where, *i*_corr, 0_ (*R*_t, 0_) and *i*_corr_ (*R*_t_) represent the corrosion currents density (charge-transfer resistance) of the WE immersed in the sample in the absence and presence of the inhibitor ECS, respectively.

Considering the contribution of the film resistance, Equation (2) would be revised to Equation (3) [27–29]:


$$IE=[(R_{t}+R_{f})-(R_{t,0}+R_{f,0})]/(R_{t}+R_{f})\times 100\%$$ (3)

where, *R*_f, 0_ and *R*_f_ represent the film resistance of the WE immersed in the sample in absence and presence of the inhibitor ECS, respectively.

### 1.5 Weight loss measurements

For hanged-up metal corrosion experiment, the metal piece is polished step by step with coarse, medium and fine sandpaper, washed successively with water and alcohol and dried to a constant weight, and accurately weighed as *w*_0_(g). Three parallel test pieces are suspended in a solution of ECS + 1mol/L HCl and allowed to stand for 6 hours. The taken-out hanged-up metals are sequentially derusted with rust removal solution, degreased with 60g/L NaOH alkali [30], washed successively with water and ethanol, and then dried to accurately weigh as *w*(g). The corrosion rate *v* (mm/y, millimeters annual) of the hanged-up metal is calculated according to the following relations [31]:


$$v\equiv (w_{0}-w)/(\rho At)=92.99(w_{0}-w)$$ (4)

where *ρ, A* and *t* respectively represent the density (7.85g/cm^3^), surface area (20.00 cm^2^) and corrosion time (6h) of the carbon steel metal; *w*_0_ and *w*, measured in grams, respectively represent the weight of the hanged-up metal before and after the hanged-up metal corrosion experiment.

Using Equation (5) can give the inhibition efficacy of weight loss experiments.


$$IE=(\nu_{0}-\nu )/\nu_{0}\times 100\%$$ (5)

where, ν_0_ and ν in mm/y, represent the corrosion rate of uninhibited and inhibited sample, respectively.

## 2 Results and discussion

### 2.1 Absorption spectra

**Fig 1:**
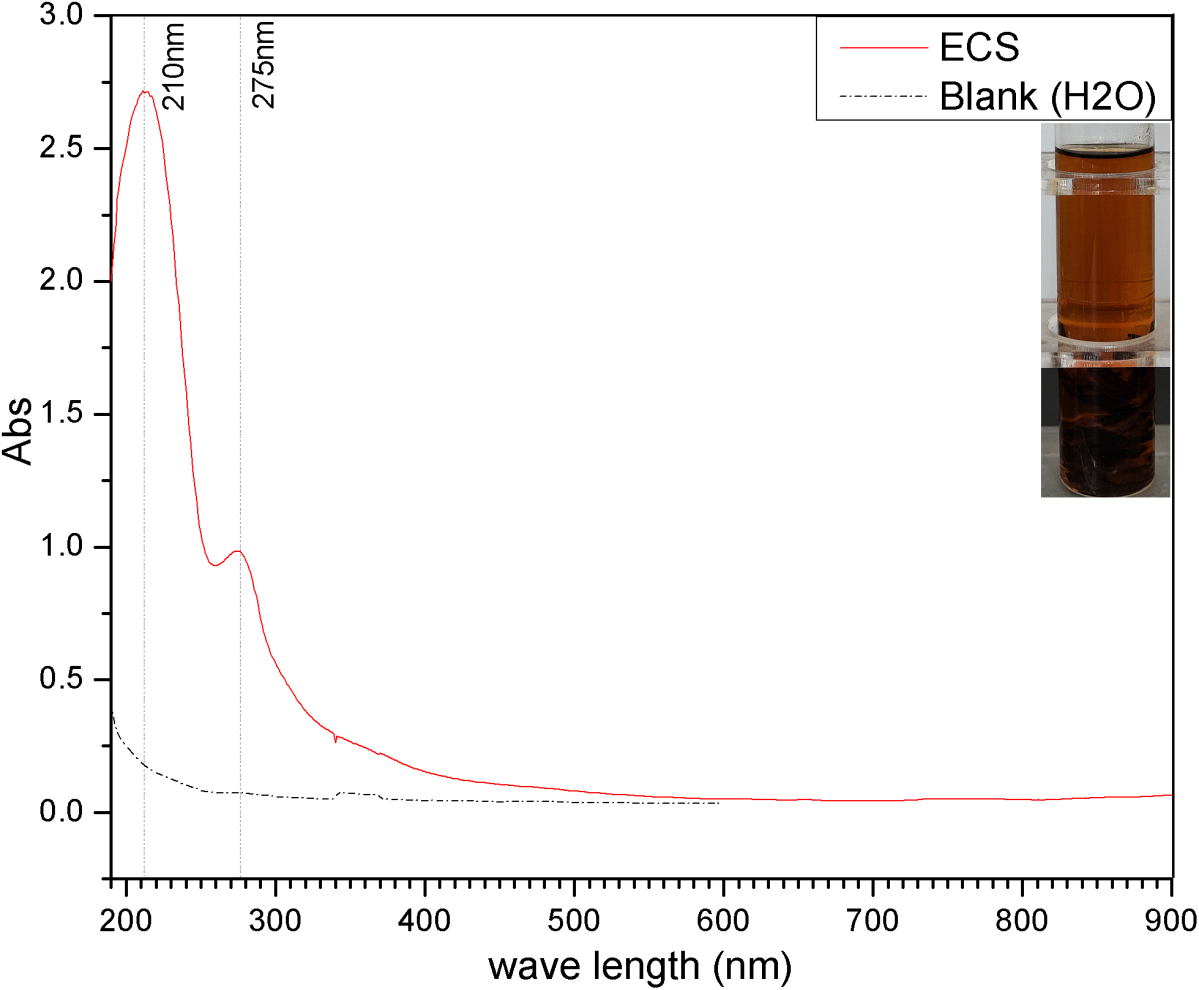
UV-vis-NIR record of the ECS (Insert: ECS sample photograph).

In addition to the highest content of cellulose of about half the weight, CS also contains water-soluble components such as flavonoids in almost glycosidic form, and some derived colorants [32]. As a free form if possible, its aglycone (previously bonded to glycosyl) is difficult to dissolve in water but easily to soluble in alcohol solvents. Fig 1 presents a photograph and a UV-vis-NIR absorption record of the ECS samples prepared from CS under alkaline conditions (pH 9) in a water-ethanol solution at 75°C.

**Fig 2:**
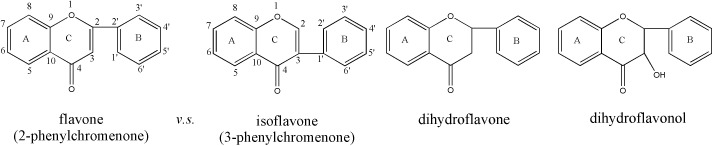
Four types of the parent/core structures of flavonoid molecules.

The inserted photograph in Fig 1 reveals that the ECS sample exhibits a brownish-yellow color in the visible light range, indicating the presence of chromophores in its molecular structure. The UV-vis record in Fig 1 shows two absorption peaks in the ultraviolet region of the ECS sample, one strong and one weak, which are consistent with the characteristic absorption of aromatic derivatives containing benzene rings. In addition, the intensity characteristics of the “stronger left and weaker right” of these two peaks aligns with the spectral absorption features of isoflavones and/or dihydroflavones/dihydroflavonols rather than flavones and/or flavonols [33]. Shown in the following Fig 2 are core structures of the just referred molecules. The increase in number of hydroxyl or methoxyl groups *etc* on the benzene ring of these structures would cause flavonoids to appear yellow even dark yellow or to undergo a red/blue shift in absorption wavelength, generating so-called I-band and II-band absorption spectral peaks dominated naturally by the electronic effect of the benzene rings.

Returning to the above Fig 1, the strong absorption peak at 210nm can be attributed to the *E*_2_ (*Elektronen*) band absorption of benzene ring, corresponding to the *π*→π* transition of electrons in the molecular orbital. While, the weak shoulder peak at 275nm is associated with the *B* (*Benzenoid*) band absorption, which arises from the electron-vibration coupling of benzene ring [34]. Here it should be noted that, compared to the *E*_2_ and *B* band absorption peaks located respectively at 200nm and 254nm of benzene itself, the corresponding peaks in the ECS spectrum exhibit a significant red shift in wavelength. This red-shift indicates that the core structures of the above-mentioned iso-/dihydro- flavones in ECS are connected to some electron-donating functional groups such as hydroxyl (−OH) and methoxyl (−OCH_3_) etc, and their *p*-*π* electron conjugation effect bring about a redshift phenomenon in the spectral absorption peak. In view of the fact that the maximum absorption of UV-vis spectrum of ECS samples is at 210nm, the 210nm wavelength is considered for its quantitative detection, and the corresponding absorbance value labeled *A*_*210*_ serves as an evaluation indicator for subsequent ECS extraction experiments.

**Fig 3:**
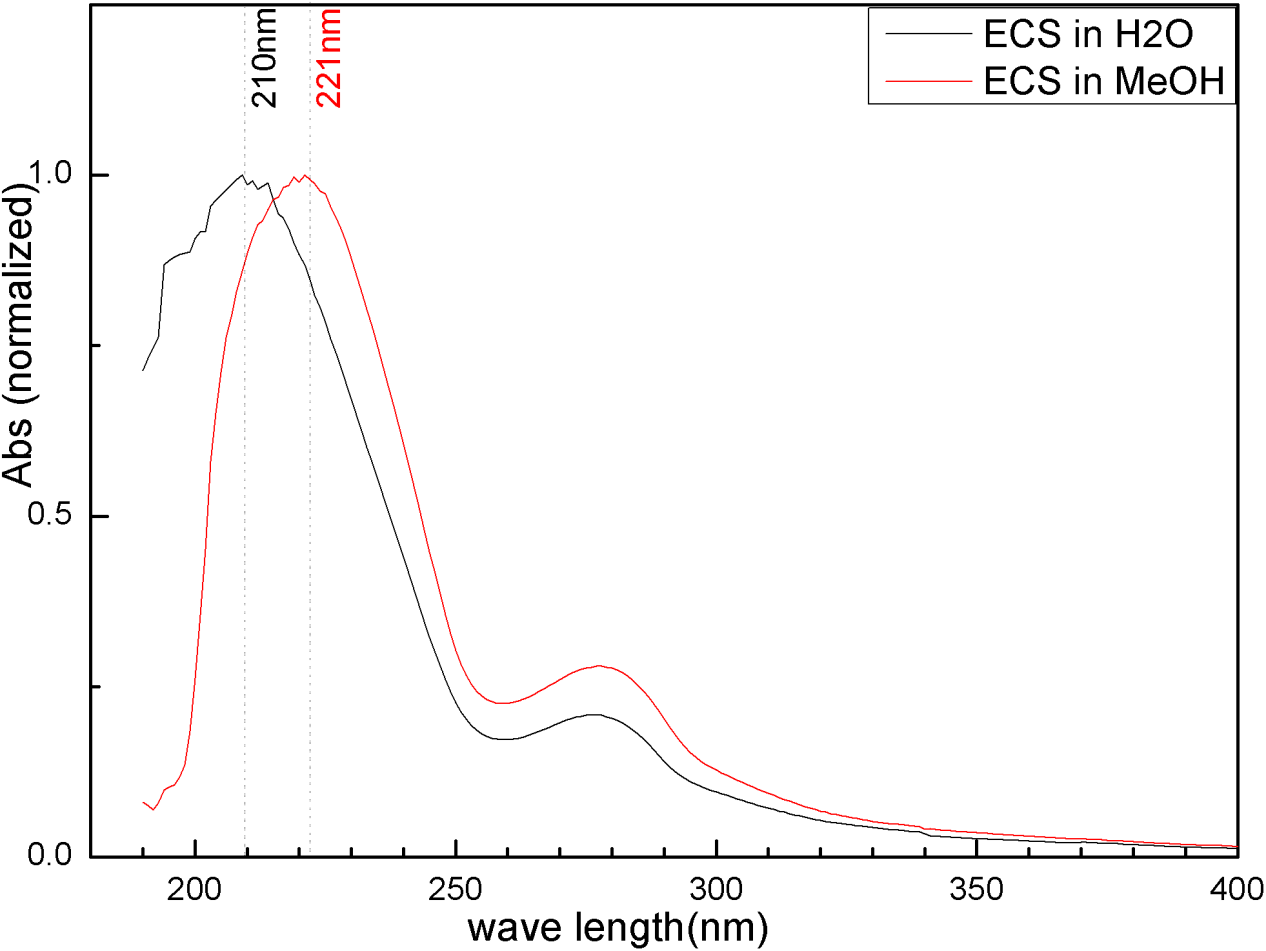
Effect of solvents (−H_2_O; −MeOH) on the UV-vis absorption spectrum of ECS.

Fig 3 illustrates the solvent effect on the UV-vis absorption of ECS samples.

When the solvent used for ECS samples is changed from methanol to water with a dielectric constant of 33.1, 80.1 [35] respectively, the maximum absorption wavelength of ECS is observed to have a blue shift from 221 to 210nm. However, such an enhancement of solvent polarity (methanol→water) ought to have resulted in a blue shift of the absorption peak wavelength applicable to the *n* → π* transition of electrons or a red shift of that applicable to the *π*→ π* transition of electrons. The reasonable explanation for the actual observation in a blue shift just mentioned is that the protonic solvent methanol and water could additionally stabilize the ground state of solute molecules in the form of hydrogen bonds, resulting in an increase in the transition energy gap. Than the solvent methanol, the highly protic water leads to a higher degree of the increase, which leads to the abnormal blue shift observed in the *E*_2_ band wavelength [36] of the ECS sample. Whereas in the *B*-band wavelength located at 275nm, almost no solvent effect is observed, which might suggest an additive contribution of *n* → π* transitions. This transition is closely related to the electronic structure of the flavonoid derivatives [37] present in CS colorants.

### 2.2 Orthogonal experiments

Several influencing factors must be considered when performing extraction according to *orthogonal experiments* (*orthog expt.*). First, the ratio of CS to solvent directly determines the concentration of the aqueous extract solution. Second, the extraction temperature influences the intensity of molecular thermal motion, thereby affecting the rate at which the target substances dissolve into the solvent. Third, ethanol can aid in the extraction of low-polarity components; however, excessive ethanol concentrations may disrupt the molecular structure and hinder extraction efficiency. Fourth, the acidity or alkalinity of the solvent affects the solubility of the target substances to be extracted. Therefore, this orthogonal experiment for ECS is designed and conducted based on an *L*_9_(3)^4^ orthogonal table, with following influence factors: mass-to-volume “concentration” of CS dispersed in water, immersion temperature, ethanol (EtOH) dosage, and pH value. The levels of these four factors are detailed in Table 1.

**Table 1:** The factors and levels of the orthogonal experiment.

Level	Factor			
	A (CS, g/L)	B (Temperature,°C)	C (EtOH, V%)	D (pH)
1	20	12	0	3
2	10	50	4	7
3	4	75	20	9

The experimental design, results, range *analysis* (*anal.*), and *verification experiments* (*verif. expt.*) are presented in Table 2, where the absorbance at 210nm (*A*_210_) of ECS sample is used as the evaluation index. The range (*R*), is calculated using Equation (6):

**Table 2:** The L_9_(3)^4^ orthogonal experiment and its results.

Task	Item	Factor				Indicator (*A*_210_)
		A	B	C	D	
Orthog expt.	1^#^	1	1	1	1	0.842
	2^#^	1	2	2	2	1.770
	3^#^	1	3	3	3	2.370
	4^#^	2	1	2	3	0.636
	5^#^	2	2	3	1	1.687
	6^#^	2	3	1	2	0.874
	7^#^	3	1	3	2	0.925
	8^#^	3	2	1	3	0.816
	9^#^	3	3	2	1	0.656
Range anal.	*ΣA*_210,1_	4.982	2.403	2.532	3.185	
	*ΣA*_210,2_	3.197	4.273	3.062	3.569	
	*ΣA*_210,3_	2.397	3.900	4.982	3.822	
	*Ā*_210,1_	1.661	0.801	0.844	1.062	
	*Ā*_210,2_	1.066	1.424	1.021	1.190	
	*Ā*_210,3_	0.799	1.300	1.661	1.274	
	Range (*R*)	0.862	0.623	0.817	0.212	
	Factor effect	A > C > B > D				
	Optimal level	A_1_	B_2_	C_3_	D_3_	
Verif.expt.	Optimal combination	(10^#^) A_1_B_2_C_3_D_3_				3.420
	Suboptimal combination	(11^#^) A_1_B_2_C_3_D_2_				3.067


$$R\;=\;{\overset{―}{A}}_{\text{210},\text{max}}\;-\;{\overset{―}{A}}_{\text{210},\text{min}}$$ (6)

where the horizontal line above A’s head means average value. Σ*A*_210,1_, *ΣA*_210,2_, and *ΣA*_210,3_ in Table 2 below, represent the sum of *A*_210_ values at the level 1/2/3, respectively.

**Fig 4:**
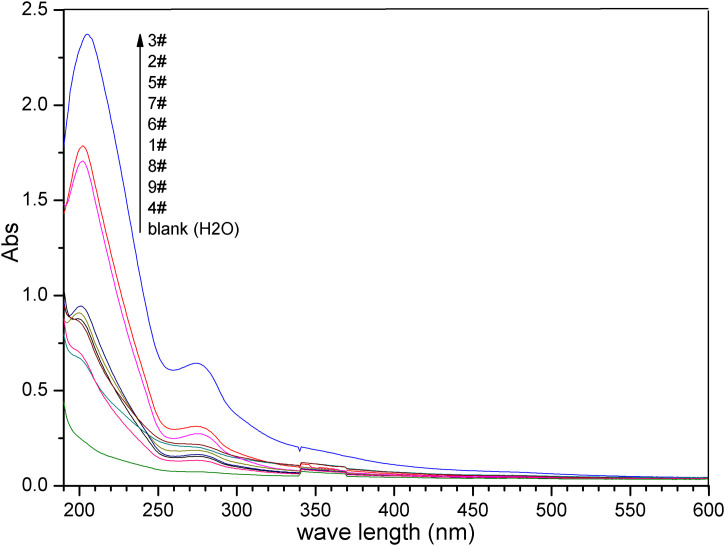
Absorption spectra of the ECS1 ~ 9^#^ samples in orthogonal experiment.

The UV-vis spectra records as the evaluation index of the experiments in Table 2 are shown in Fig 4. Its visual inspection reveals that the experiment 3^#^ is the most effective among the nine orthogonal experiments, but it is a local and not necessarily a global optimum according to statistical theory.

**Fig 5:**
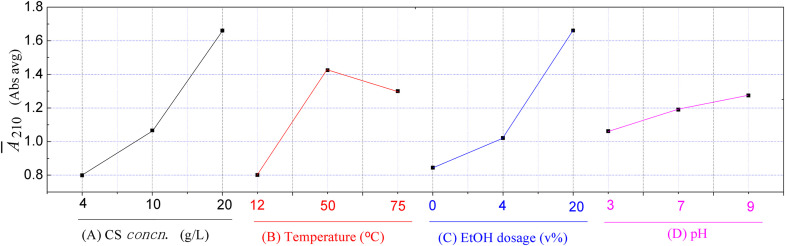
Trend charts of the effects of the four factors in orthogonal experiment for ECS.

It is evident from Table 2 that the ECS samples with higher absorbance values are obtained with higher concentrations of CS or/and higher dosages of ethanol, while the soaking temperature and pH value have a relatively minor impact on extraction efficiency. The trend of each factor’s influence on extraction efficiency is illustrated in Fig 5. It can be observed that the change in *Ā*_210_ with pH value is minimal, indicating that pH value has little effect on extraction efficiency. In contrast, variations in CS concentration or ethanol dosage result in significant changes in *Ā*_210_ values, suggesting that these factors have a substantial impact on extraction efficiency.

The results of range analysis listed in Table 2 indicate that the globally optimal combination of factors for this orthogonal experiment is A_1_B_2_C_3_D_3_, which is used to conduct experiment 10^#^ for verification. Additionally, considering the small difference in range analysis results when factor D is at the level 2 and 3, the globally suboptimal combination A_1_B_2_C_3_D_2_ is also tested as experiment 11^#^ for verification. Results of the two verification experiments, as shown in Table 2, demonstrate that the ECS sample from experiment 10^#^ meets expectation better than that from experiment 11^#^. Therefore, the ECS sample from experiment 10^#^ (ECS10^#^) is selected for all subsequent studies.

### 2.3 Tafel polarization curves

**Fig 6:**
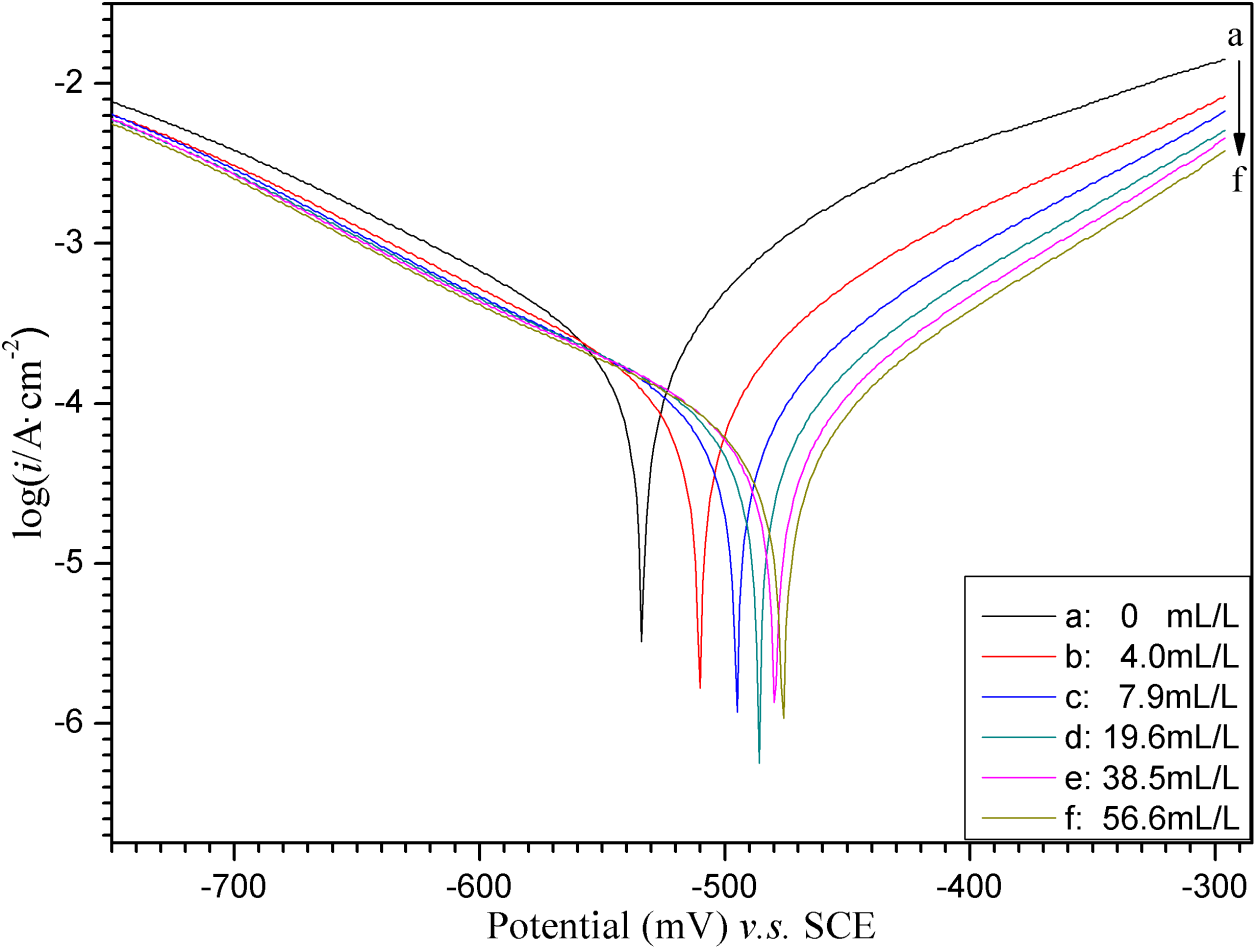
Tafel curves of “ECS (various *concn.* a→f)+1mol/L HCl+WE”.

Fig 6 shows the Tafel polarization curves of carbon steel WE with varying ECS concentrations in a 1mol/L hydrochloric acid solution.

What can be seen at a glance in the above figure is that the increase in concentration of ECS leads to a remarkably positive shift in self-corrosion potential (*E*_*corr*_) of carbon steel in the corrosive medium, while the corrosion current density (*i*_*corr*_) in the Tafel region decreases significantly compared to the blank solution without ECS. The former indicates that ECS improves the thermodynamic stability of carbon steel in acidic media, while the latter indicates that ECS inhibits the corrosion rate of carbon steel in acidic media. This at least roughly indicates that ECS could serve as an effective corrosion inhibitor for carbon steel in acidic media.

The Tafel polarization parameters corresponding to Fig 6 are summarized in Table 3, where, the “ *η*= *a*+*b*log*i”,* log denotes the base-10, serves as the universal form of the Tafel formula.

**Table 3:** Electrochemical parameters of the Tafel curves.

ECS *concn*. (mL/L)	*E*_*corr*_ (mV *vs* SCE)	*b*_c_ (mV/dec)	*b*_a_ (mV/dec)	*R*_p_ (Ω)	*i*_corr_ (×10^−^^5^A)	*IE* (%)
0	−534	−127.0	123.9	90.1	30.26	–
4.0	−510	−129.2	117.8	170.6	15.71	48.08
7.9	−495	−134.8	107.4	235.0	11.06	63.45
19.6	−486	−140.8	105.5	278.1	9.429	68.84
38.5	−480	−144.6	103.5	313.5	8.367	72.35
56.6	−476	−146.2	103.5	349.8	7.531	75.11

As shown in the table above, the 58mV positive shifts of *E*_*corr*_ from −534mV to −476mV after adding the inhibitor ECS, although not reaching the critical value of 85mV, is still appreciably close to it as well as prominently exceeds the error range of up to 30mV potential measurement, indicating that this shift cannot be explained solely by the blocking effect of the corrosion inhibitor on surface of electrode, and suggesting the contribution of the anodic type control of ECS to corrosion inhibition.

The data in detail presented in the Table 3 specifically indicate that an increase in concentration of ECS in 1mol/L HCl solution leads to not only a positive shift in *E*_*corr*_, but also an increase in polarization resistance (*R*_*p*_), and hence the corrosion current density (*i*_*corr*_) significantly decreases, which means that the corrosion inhibition efficiency (*IE*) is correspondingly improved. These results demonstrate that the higher the dosage of ECS, the better the corrosion inhibition effectiveness. At an ECS dosage of 56.6mL/L, the *i*_*corr*_ decreases to 7.531×10^−5^A/cm^2^ against 30.26×10^−5^A/cm^2^ of the blank, accordingly achieving a corrosion inhibition efficiency of 75.11%, which is approximately the same as the reported [9,10]. It must be noted that such an *IE* calculated based on *R*_*p*_ here does not have a very reliable prerequisite. This is due to the inability to correct the ohmic resistance of the sample solution and the possible changes in Tafel slope before and after the addition of corrosion inhibitors, resulting in a certain degree of error in the obtained *IE*. What is also evident from the Table 3 is that the Tafel *c*athodic or *a*nodic slope of the blank solution represented by *b*_*c*,0_ or *b*_*a*,0_ respectively is close to 120mV/decade, which is consistent with the value of *b* for most metals at room temperature. And moreover, the Tafel *b*_*c*_ and *b*_*a*_ of the sample solutions containing ECS are both different from those of the blank solution, which means the ECS simultaneously affects the kinetic parameters of the cathodic and anodic electrode reaction processes of carbon steel in acidic media. Compared with the Tafel slope value |*b*_c,0_| or |*b*_a,0_|, the |*b*_*c*,ECS_| or |*b*_*a*,ECS_| undergoes continuous changes of increasing and decreasing respectively, indicating that the ECS here as a corrosion inhibitor can be assigned to a mixed type inhibition.

The inhibition mechanism of ECS is likely to be the formation of a protective film covering the anode area on the surface of carbon steel. This is certainly attributed to the components rich in hydroxyl (−OH) and oxygen atoms in ECS, which could be the colorants, flavonoid glycosides, and some of their hydrolysis products such as aglycones, polyphenols, and glycosyls etc. The oxygen atoms possess high electronegativity and lone pairs of electrons. These lone pairs can form a certain degree of coordinate bonds with the vacant *d*-orbitals of iron (Fe) atoms on the steel surface, resulting in chemi-/physi- sorption and the formation of a protective film. This film reduces the reactivity of Fe atoms with H^+^ ions in the acidic environment, thereby inhibiting corrosion.

In addition, the formed film also partially covers the cathode active sites, increasing the difficulty of cathode reaction (reduction of H^+^), resulting in an increase in the cathodic Tafel slope. Then the ECS can be considered a typical mixed-type corrosion inhibitor with some cathodic and prominent anodic control.

The decreases in the |*b*_*a*,ECS_| does not indicate that the corrosion inhibitor ECS promotes the anode reaction, but rather reveals its unique mechanism of action. The most reasonable explanation is the “selective coverage and active site concentration” model. In the solution containing corrosion inhibitors, the most active sites are “sealed”, and the anodic dissolution reaction is forcibly transferred to those sites that are not or weakly protected and slightly less activity. From the perspective of the electrode as a whole, in order to generate a certain apparent anode current, very rapid reactions need to occur at those limited active points, and so the local real current density is very high at these active points. This is manifested as the generation of larger currents at lower overpotentials, which is macroscopically reflected as a decrease in the anode Tafel slope. But macroscopically, due to the fact that the protected area is much larger than the active site, the overall apparent anode current (*i*_*corr*_, i.e., overall corrosion rate) still sharply decreases.

### 2.4 EIS results

**Fig 7:**
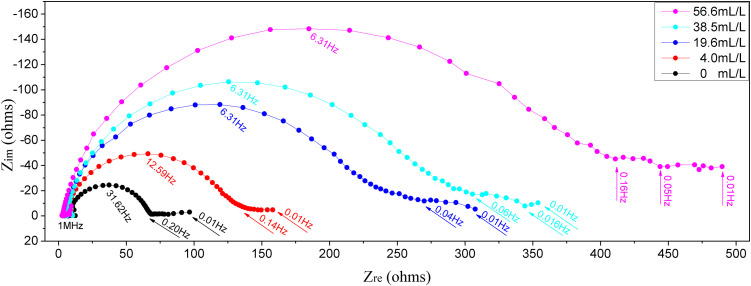
Nyquist plots of EIS for “ECS (various *concn*.)+1mol/L HCl”.

**Fig 8:**
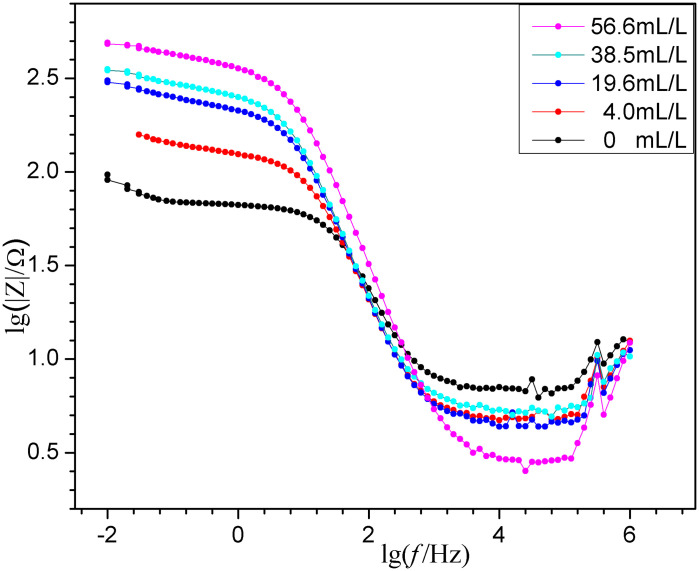
Bode |Z| plots of EIS for “ECS (various *concn*.)+1mol/L HCl”.

**Fig 9:**
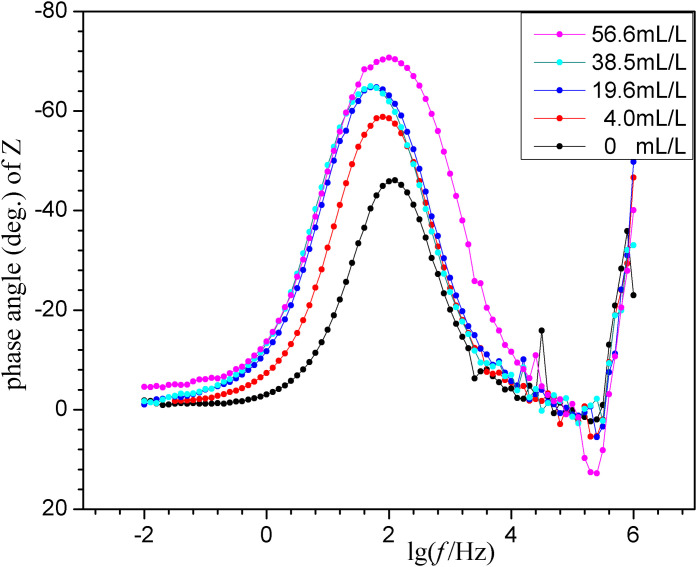
Bode phase angle plots of EIS for “ECS (various *concn*.)+1mol/L HCl”.

EIS is performed on carbon steel in a 1mol/L HCl medium with varying ECS concentrations, applying a small-amplitude AC voltage. The results are presented in Figs 7-9.

Usually due to the negligible influence of mass-transfer during the corrosion process in acidic solutions, the Nyquist plots (with same scale on the vertical and horizontal axes) shown in Fig 7 show their short segments of diffusive mass-transfer in the low-frequency region. Also shown is that the leftmost starting points of the plots are close to the horizontal axis origin, indicating negligible *solution resistance* (*R*_*s*_), consistent with the *R*_*s*_ values listed in following Table 4 derived from the equivalent circuit simulation. While, there are three important observations in Fig 7 as follows. The “widened and raised semi-circles *v.s.* the blank” are related to the suppression of electron transfer during the corrosion processes. The “two time constants” at higher concentrations of ECS is evident or clearly in Fig 9 for a dual-layer structure at the interface, confirming the ECS forms a distinct adsorption *film* (R_f_) separate from the charge *transfer* process (R_t_) at the metal surface. The “correlative with ECS concentration”, shows the semicircle diameter for the blank is significantly smaller than those for the samples containing ECS, and the diameters have a positive trend with greater doses of ECS. This demonstrates that ECS effectively inhibits electron transfer at the electrode interface.

In the ordinary way, samples with higher *impedance modulus* (*|Z|*) usually mean higher corrosion resistance. The Bode plots in terms of the *|Z| v.s.* frequency are shown in Fig 8. It reveals that the |Z| values in the low-frequency region increases with ECS concentration, further confirming the enhanced corrosion inhibition effect of ECS samples. The Bode plots in terms of the phase angle *v.s.* frequency are shown in Fig 9. It exhibits a relatively weak peak at low frequency and a clear strong peak at higher frequency, indicative of an adsorption film formed by ECS on the carbon steel surface. The Bode |Z| and phase angle plots in the presence of the ECS are greater than those of the blank, confirming its better protective properties. The blank's plot in Fig 9 indicates the presence of an active higher-frequency relaxation associated with rapid surface dissolution or rough double layer in the blank sample. Distinct from it, the ECS brings a significant negative shift in phase angle peak from −46.1^o^ of blank to −70.7^o^ of samples' maximum, indicating that the resistance component controlling the relaxation process become extremely large, *i.e*. suggesting that the original active sites of the blank are covered and replaced by a molecular film with high resistance and low capacitance characteristics. That is, the corrosion inhibitor molecules, through strong adsorption, transform the “active interface” that originally corresponded to rapid ion exchange/dissolution into a “barrier interface” with high resistance to charge/ion transport. It no longer primarily reflects dissolution kinetics, but rather the transport kinetics of reactants/products across this high resistance corrosion inhibitor adsorption film. Equivalent circuits can help explain this.

The EIS parameters derived from a dual-time-constant equivalent circuit simulation/fitting of Fig 7-9 are summarized in Table 4.

**Table 4:** Electrochemical parameters of the EIS results.

ECS *concn.* (mL/L)	*R*_*s*_ (Ω)	*CPE*_*f*_		*R*_*f*_ (Ω)	*CPE*_*dl*_		*R*_*t*_ (Ω)	*IE* (%)
		Y_0_(sec^n^Ω^−1^) [14,20]	n		Y_0_(sec^n^Ω^−1^)	n		
0	6.934	1.618 × 10^−4^	0.8747	60.71	20.710 × 10^−2^	0.8656	17.17	/
4.0	4.882	1.661 × 10^−4^	0.8895	118.3	2.795 × 10^−2^	0.7037	28.07	46.79
19.6	4.594	1.655 × 10^−4^	0.8859	215.4	1.998 × 10^−2^	0.7038	72.41	72.94
38.5	5.370	1.634 × 10^−4^	0.8845	259.1	1.880 × 10^−2^	0.7184	77.31	76.85
56.6	2.899	0.9733 × 10^−4^	0.8954	333.7	0.7471 × 10^−2^	0.4323	216.6	85.85

where, *R*_*t*_ and *R*_*f*_ respectively represent the charge-transfer resistance and film resistance of the material. As shown in the table, at ECS concentrations ranging from 4.0 to 56.6 mL/L the calculated corrosion *IE* reaches approximately 50 to 85%, with a reasonable difference of around 10% from the Tafel curve results. The *R*_*t*_ and *R*_*f*_ both increase positively with ECS concentration, reflecting the “*R*_*t*_+*R*_*f*_”-jointly suppressed corrosion reaction rates of carbon steel as electrode materials. This indicates that ECS, as a corrosion inhibitor, exhibits a synergistic effect of both negative catalysis and geometric coverage/blocking. While also is seen that *R*_*f*_ is several times higher than *R*_*t*_ at low to medium concentrations of ECS, and the two are only comparable at higher concentration of ECS, indicating that the initial addition of ECS is mainly based on adsorption film, and then the negative catalytic effect is roughly equal to the coverage effect in the later stage.

Due to chemical reactions and variations of current or potential on the surface of electrode, the assumption of a uniformly active electrode is generally invalid, resulting in a observation of dispersion effect in the time constant of *EIS*, that is, some degree of deviation from pure *Capacitive elements* (*C*). Like the *C*_*dl*_ (*double layer*), the *constant phase element* (*CPE*) is also an admittance (*Y*) caused by the non-*Faraday* process, with an impedance of the following [14,38]:


$$Z_{CPE}=\text{1}/[Y_{\text{0}}(j\omega {)}^{\text{n}}]$$ (7)

where $j=\sqrt{-1}$. The two important parameters of *CPE*, *n* and *Y*_*0*_, are also listed in Table 4, which respectively describe the deviation from the ideal double-layer capacitance behavior (dispersion index) and the charge storage capacity of the electrode surface (including capacitance information). As can be seen, all *n* are less than 1, reflecting the roughness, unevenness, and energy dissipation of the electrode surface. Especially the index *n* value of the film layer is also less than 1 but between (0.8, 0.9), indicating noticeable non-uniformity in the ECS-covered film on the electrode surface, which is consistent with the “selective coverage and active site concentration” model inferred from the Tafel results in the previous section. The *Y*_*0*_ of *CPE*_*dl*_ significantly decreases with the addition of ECS, suggesting that the adsorbed ECS inhibitor weakens the electrical double layer at the electrode-solution interface.

The equivalent circuit fitting results also show that the adsorption layer resistance (R_f_) significantly increases from 60.71 to 333.7ohms while the capacitance (C_f_) decreases relatively from 1.618 to 0.9733. This is also consistent with the systematic change in the peak frequency of the capacitance arc observed in Fig 7: from 31.62Hz in the blank solution to 12.59Hz, and stable at 6.31Hz at high concentrations of ECS. This decrease in characteristic frequency directly indicates a significant increase in the time constant of the interface dominant relaxation process. It dynamically confirms that the adsorbed ECS film layer acts as a transport barrier, greatly delaying the transfer process of interface charges/ions. The flattening of characteristic frequencies at high concentrations of ECS indicates that the adsorption layer structure has reached saturation coverage within this concentration range, and the interface dynamics have entered a stable state.

**Fig 10:**
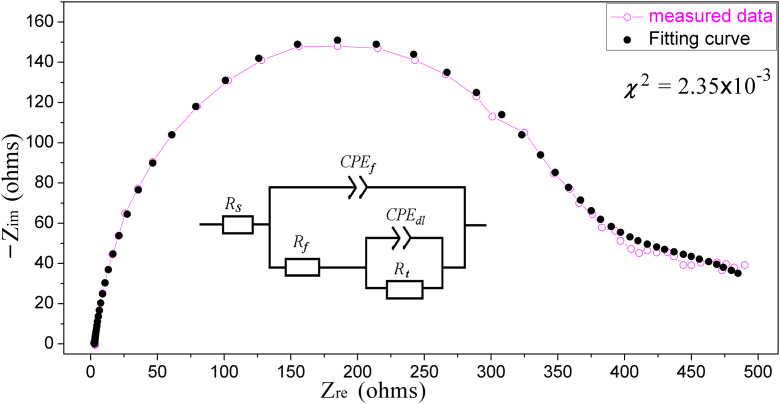
Equivalent circuit (insert) and Nyquist plots of the fitted (●) *v.s.* measured data (−o−) of ECS (56.6mL/L) sample.

To illustrate, the Nyquist plot for the 56.6mL/L ECS sample is simulated using the equivalent circuit shown in Fig 10 (insert) [39,40]. This circuit replaces the ideal capacitor with a CPE to account for non-Faradaic process on electrode surface and the deviation of the EIS capacitance arc from a perfect semicircle, as evidenced by the dispersion index *n* with values less than 1 in Table 4. The fitted Nyquist plot shown in Fig 10 closely matches the experimental data, with a statistical chi-square value (*χ*^2^ = 2.35×10^−3^) confirming excellent agreement between the model data and measurements data.

### 2.5 Adsorption isotherms

In *aqueous* (*aq*) corrosion systems, solvent water molecules preferentially adsorb onto metal surfaces. So-called *adsorption*(*ads.*)-type corrosion inhibition occurs when organic corrosion *inhibitor* (*Inh*.) molecules replace adsorbed water molecules to establish adsorption equilibrium, governed by the equilibrium constant *K*. This process can be expressed as following Equation (8):


$${Inh}_{\left(aq\right)}\;+\;{xH}_{\text{2}}O_{\left(ads\right)}\;\overset{\text{K}}{⇌}\;{Inh}_{\left(ads\right)}\;+\;{xH}_{\text{2}}O_{\left(aq\right)}$$ (8)

where *x* as size parameter, represents the number of water molecules displaced by one inhibitor molecule.

To fit experimental data, the relationship between surface coverage (*θ*) and inhibitor *concentration* (*c*) can be described by various adsorption isotherm models, including Langmuir, Freundlich, Temkin, Frumkin, Flory-Huggins, and so on [41]. These models are defined by following equations [42] and their linear forms in Table 5:

**Table 5:** Five isotherm adsorption models and their equations.

Expression	Isothermal adsorption equation	The linear form of “*y= kx+b”*	No.
Langmuir isotherm	$\theta /(1-\theta )=K_{ads}c$	$\theta /(1-\theta )=K_{ads}c$	(9)
Freundlich isotherm	$\theta =K_{ads}c^{1/n}$	$\log\theta =(1/nlogc+\log K_{ads}$	(10)
Temkin isotherm	$\exp(f\theta )=K_{ads}c$	$\theta =(2.303/flogc+(2.303/f)\log K_{ads}$	(11)
Frumkin isotherm	$[\theta /(1-\theta )]e^{-2a\theta }=K_{ads}c$	$\log(\theta /[(1-\theta )c])=(2a\log etheta+\log K_{ads}$	(12) [22]
Florry-Huggins isotherm	$\theta /[x(1-\theta {)}^{x}]=K_{ads}c$	$\log(\theta /c)=x\log(1-\theta )+\log(xK_{ads})$	(13) [20]

where *K_ads_* is the adsorption equilibrium constant, *n* is a correction coefficient, *f* is a configurational factor dependent essentially on the physical model, and *a* is the interaction coefficient (positive for attraction, negative for repulsion; *a* = 0 reduces Frumkin to Langmuir).

It is generally assumed that corrosion reactions only occur at active sites not covered by corrosion inhibitor molecules, so the corrosion *inhibition efficiency* (*IE*) obtained from corrosion experiments can be used to calculate/estimate/deﬁne the surface coverage (*θ*) using:


θ=IE(%)/100 (14)

**Fig 11:**
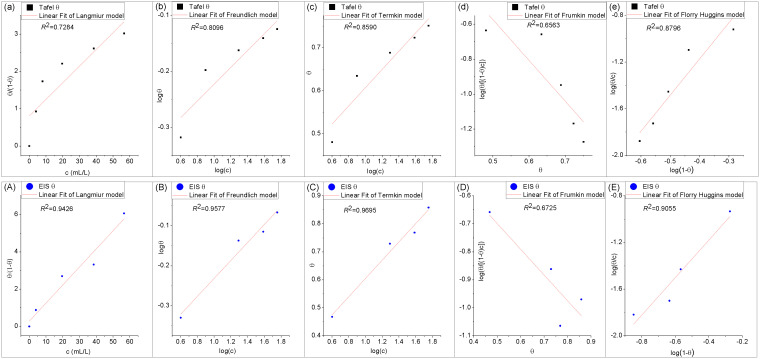
Linear fitting of isothermal adsorption models of ECS adsorbed on WE in 1M HCl at room temperature (5°C).

Experimental data are linearly fitted to the above five adsorption isotherm models. Fig 11 compares the linear fitting performance based on EIS data and Tafel data. Both the visual inspection and the quantitative *goodness-of-fit* (*R*^2^) analyses demonstrate that the linear fitting performance of the former datasets (Fig 11A–E) is superior to that of the latter datasets (Fig 11a–e). The Temkin isotherm (Fig 11C) exhibits the highest coefficient of determination (*R*^2^ = 0.9695) for the ECS adsorption, indicating its suitability for describing medium coverage (*θ* = 0.2 ~ 0.8), consistent with electrochemical results. In contrast, the Langmuir model (*R*^2^ = 0.9426), which assumes ideal monolayer adsorption without intermolecular interactions, shows somewhat poor agreement with the experimental data.

The adsorption equilibrium constant (*K*_*ads*_*)* derived from the Temkin model is listed in Table 6.

**Table 6:** Linear fitting parameters derived from the Temkin isothermal adsorption model of Fig 11C.

Temkin	linear fitting equation	slope	intercept	*R* ^2^	*K*_*ads*_ (L/mol)
model	θ=(2.303/flogc+(2.303/f)logK	2.303/*f*	(2.303/*f)*log*K*		
fitting	θ=0.3270logc+0.2783	0.3270	0.2783	0.9695	2254.50

As the most important thermodynamic adsorption parameter, the change in standard free energy of adsorption ($\Delta G_{ads}^{0}$) of ECS on carbon steel surface, can be calculated using Equation (15) [43]:


$$\Delta G_{ads}^{0}=-RT\ln(55.5K_{ads})$$ (15)

where 55.5 is the molar concentration (mol/L) of water molecule at metal/solution interface.

The calculated $\Delta G_{ads,278K}^{0}$≈−27.13 kJ/mol, which is negative, indicating a spontaneous adsorption of the ECS on carbon steel surface. Furthermore, the value falls within the range of (−40, −20) kJ/mol representing the critical values for distinguishing chemical adsorption *via* electron sharing or covalent bonding and physical adsorption *via* electrostatic interactions respectively, suggesting a mixed adsorption mechanism involving both physical and chemical interactions of ECS on carbon steel surface.

### 2.6 Weight loss results

What is most important in practical applications is whether the actual corrosion rate matches the standard requirements. As a test to simulate actual corrosion, static weight loss results demonstrate that while the corrosion rate of the uninhibited blank sample gives 5.27mm/y, the addition of ECS itself as a corrosion inhibitor at concentration of 19.6 or 56.6mL/L reduces the corrosion rate of carbon steel to 1.30 or 1.21mm/y, which well meets the limit of 2.23mm/y of the corrosion rate requirement specified by the current national standard for laboratory verification [44]. Accordingly, the actual inhibition efficiency of the ECS here is given as 75.33% or 77.04% by Equation (5), roughly equivalent to the electrochemical data provided in the previous sections.

## 3 Conclusions

As a new candidate for corrosion inhibitor as well as a new application of food waste CS, the optimizing-obtained ECS sample in this manuscript reflects eco-friendly and cost-effective characteristics of green corrosion inhibitors as well as the principle of waste resource recycling. For the sample, UV-visible absorption provides its electronic transition type, an orthogonal array investigates the four influencing factors of its preparation, and the Tafel curve and EIS give its electrochemical behaviors. The Temkin-modeled mixed adsorption of the ECS on the surface of carbon steel in strongly acidic environment exhibits a feature of mixed inhibition dominated by anodic control. Nearly 80% as the alone ECS-induced *IE* is acceptable, and the actual corrosion rate inhibited to around 1.21mm/y is sufficient to meet the national standard. This finding highlights its considerable potential for industrial and engineering applications in acid-pickling and corrosion protection of carbon steel equipment. In future, the acquisition of pure samples from ECS and the physical chemistry processes that affect metal corrosion are worth investigating in depth.
